# MyoSight—semi-automated image analysis of skeletal muscle cross sections

**DOI:** 10.1186/s13395-020-00250-5

**Published:** 2020-11-16

**Authors:** Lyle W. Babcock, Amy D. Hanna, Nadia H. Agha, Susan L. Hamilton

**Affiliations:** Department of Molecular Physiology and Biophysics, Baylor College of Medicine, One Baylor Plaza, Houston, TX 77030 USA

**Keywords:** Duchenne muscular dystrophy, Soleus, Cross-sectional area, Fiber type, Myonuclei, Central nuclei, FIJI Plugin

## Abstract

**Background:**

Manual analysis of cross-sectional area, fiber-type distribution, and total and centralized nuclei in skeletal muscle cross sections is tedious and time consuming, necessitating an accurate, automated method of analysis. While several excellent programs are available, our analyses of skeletal muscle disease models suggest the need for additional features and flexibility to adequately describe disease pathology. We introduce a new semi-automated analysis program, MyoSight, which is designed to facilitate image analysis of skeletal muscle cross sections and provide additional flexibility in the analyses.

**Results:**

We describe staining and imaging methods that generate high-quality images of immunofluorescent-labelled cross sections from mouse skeletal muscle. Using these methods, we can analyze up to 5 different fluorophores in a single image, allowing simultaneous analyses of perinuclei, central nuclei, fiber size, and fiber-type distribution. MyoSight displays high reproducibility among users, and the data generated are in close agreement with data obtained from manual analyses of cross-sectional area (CSA), fiber number, fiber-type distribution, and number and localization of myonuclei. Furthermore, MyoSight clearly delineates changes in these parameters in muscle sections from a mouse model of Duchenne muscular dystrophy (mdx).

**Conclusions:**

MyoSight is a new program based on an algorithm that can be optimized by the user to obtain highly accurate fiber size, fiber-type identification, and perinuclei and central nuclei per fiber measurements. MyoSight combines features available separately in other programs, is user friendly, and provides visual outputs that allow the user to confirm the accuracy of the analyses and correct any inaccuracies. We present MyoSight as a new program to facilitate the analyses of fiber type and CSA changes arising from injury, disease, exercise, and therapeutic interventions.

## Introduction

Accurate measurements of cross-sectional area (CSA), fiber-type distribution, and myonuclei number and location provide critical information needed to evaluate the consequences of disease and injury and to evaluate the efficacy of therapeutic interventions and/or exercise in improving skeletal muscle function [1]. These measurements, when completed manually, are time consuming and prone to user error and bias. The tedious nature of manual analysis typically leads to a low number of muscle fibers being analyzed, potentially affecting overall accuracy in research and clinical conclusions.

There are several programs currently available for histological segmentation of muscle fibers to quantify CSA, fiber-type distribution, perinuclei (nuclei along the perimeter of fiber), and central nuclei [2–11]. Several of these programs operate on the freely available platform FIJI and are compatible with Apple computers running Mac OS X (Mac) and personal computers running Windows (PC) operating systems [5–7, 10]. Other programs are stand-alone and compatible only with a PC [8, 11]. While several programs accept the original file format (Bio-Format images saved directly from proprietary life sciences software) where scaling information is embedded in the file [6], others require TIFF formats, where scaling information must be entered manually [5, 7, 8, 10, 11]. Two of the programs are semi-automated and allow for manual corrections during the analysis [10, 11]. Other programs are fully automated and do not allow user input during the analysis [6, 8]. While all of the current programs are freely available, they display variability in terms of output, ease of use, and accuracy in identifying muscle fibers and their characteristics.

To obtain accurate CSA measurements, it is critical that the program precisely identifies the membrane borders of individual muscle fibers. The common method for identifying membrane border is immunofluorescent (IF) staining of membrane proteins, usually with antibodies to laminin or dystrophin [6, 8, 10, 11]. The algorithm used by each program to detect membrane borders and the quality of the immunofluorescent staining affect the accuracy of the results. While full automation has the benefit of eliminating user bias, it prevents error correction and may reduce the accuracy of the final quantification. Semi-automation can improve the accuracy of the results but increases the time required to perform the analysis [10, 11]. A program that combines accurate, customizable semi-automation with a user-friendly interface that minimizes the time and difficulty of post-analysis corrections is needed.

In this manuscript, we describe a new semi-automated image analysis tool called MyoSight, which operates as a FIJI plugin. MyoSight is designed to optimize some features of the available image analysis software, including ease of use, availability to the researcher, accuracy in identifying/measuring fiber borders, CSA, fiber-type, and the number of central and perinuclei (nuclei along the perimeter) per fiber. This new program introduces input features that allow user-guided optimization of the parameters to analyze an image. These features, combined with the ability to manually correct incorrect fiber assignments, contribute to MyoSight’s accuracy. MyoSight accepts TIFF and JPEG files, as well as Bio-Format images generated by the image acquisition software. We describe and make available MyoSight to the skeletal muscle community and compare its accuracy, ease of use, and efficiency to traditional manual methods.

## Methods

### Collection and preparation of muscle samples

Soleus muscles from 16-week-old wild-type (WT, *n* = 3) and mdx (*n* = 3) mice on a C57bl/10 background were used to test the program’s ability to recognize variability in myofiber morphology. All mice were anesthetized using isoflurane and euthanized by cervical dislocation. The soleus muscles were dissected and frozen in optimal cutting temperature (OCT) medium using liquid nitrogen-cooled isopentane and stored at − 80 °C. Muscle samples were sectioned at 10-μm thickness, mounted on charged glass slides, and stored at − 20 °C. All experimental protocols using animals were approved by the Baylor College of Medicine Institutional Animal Care and Use Committee.

### Immunofluorescence imaging

Image analysis programs rely on immunofluorescent labeling. We provide a reproducible and reliable staining protocol for use with the analysis programs (supplemental material). The combination of laser scanning confocal microscopy with spectral imaging and linear unmixing permits differentiation of up to five channels in a single sample. Using these techniques allows simultaneous imaging of cell membranes, identification of four fiber-types as well as hybrid fibers, and quantitation of nuclei. All primary and secondary antibodies used in our analyses are listed in Table 1. Frozen muscle cross-sections were fixed in 4% paraformaldehyde for 5 min, washed twice in phosphate-buffered saline (PBS), and incubated in 50 mM sodium hydroxide for 30 min. The sections were washed twice in PBS and incubated for 5 min in 0.1% TX-100 in PBS for permeabilization. All subsequent wash solutions use PBS with 0.05% TX-100. The washed sections were incubated in 4% heat-inactivated goat serum in PBS for 1 h followed by incubation in primary antibodies diluted in blocking buffer, overnight at 4 °C. Samples were washed several times over the course of 30 min and incubated for 2 h at room temperature with secondary antibody. To remove secondary antibody, samples were washed several times with PBS over 30 min. After 5 min incubation in DAPI for nuclei staining, the samples were washed several times with PBS without TX-100, mounted in Fluoromount G (Southern Biotech) with a glass coverslip, and sealed with clear nail polish. A detailed protocol is available in the MyoSight instruction manual provided in the supplemental material.

**Table 1:** **Antibody Cocktails**

	Primary antibody	Secondary antibody
**Laminin** (membrane borders)	Abcam, ab11575 Rabbit IgG 2 μg/ml	ThermoFisher, A-11035 Goat Anti-Rabbit IgG Alexa Fluor 546 1 μg/ml
**MHC I**	DSHB BA-F8 Mouse IgG2b 20ug/ml	ThermoFisher, A-21242 Goat Anti-Mouse IgG2b Alexa Fluor 647 10 μg/ml
**MHC IIa**	DSHB SC-71 Mouse IgG1 20 μg/ml	ThermoFisher, A-21121 Goat Anti-Mouse IgG1 Alexa Fluor 488 10 μg/ml
**MHC IIb**	DSHB BF-F3 Mouse IgM 20 μg/ml	ThermoFisher, A-21044 Goat Anti-Mouse IgM Alexa Fluor 594 10 μg/ml
**Myonuclei**	Invitrogen D3571 DAPI 1:500 dilution	

Primary and secondary antibody cocktail combinations for immunofluorescent labeling of laminin, MHC I, MHC IIa, MHC IIb, and myonuclei

The excitation/emission spectra of Alexa Fluor 546, 594, and 647 fluorophores have some overlap which necessitates doing a lambda scan and spectral unmixing using the microscope’s software. For this process, WT samples were stained with either DAPI, laminin/546, MHC I/647, MHC IIa/488, or MHC IIb/594 on separate slides for their spectral array to be determined by the microscope’s software. For optimal image acquisition (explained in detail below) experimental samples treated with only secondary antibody (subjected to the IF staining in the absence of primary antibody) were used as controls for nonspecific binding imaged concurrently with samples exposed to both primary and secondary antibody.

### Image acquisition

Immunofluorescent-stained cross sections were imaged on a Zeiss 880 laser-scanning confocal microscope with the ZEN Black imaging software. All experimental images were taken at × 10 magnification with 1024 × 1024 pixel resolution. IF labeling of more than four proteins of interest requires a modified acquisition process since the excitation and emissions spectra of the secondary antibodies overlap. First, the spectral array of each individual label was assessed separately using the microscope’s software. Once this was completed, a lambda scan was used to record the excitation spectra from all labels in an experimental image simultaneously. Finally, each label was separated into its own channel by the spectral unmixing functions of the microscope’s software.

To minimize nonspecific background, secondary antibody-only controls were used to optimize the imaging parameters. Laser power, gain, and offset on the microscope’s software were adjusted so that the image produced no signal from secondary-only controls. These parameters were then used to image experimental samples (exposed to both primary and secondary antibodies) where robust fluorescent signals were evident for all fluorophores. To obtain an optimal signal from the target protein without overexposure, we optimized the laser and adjusted microscope gain to maximize the brightness of the positive signal without saturating the signal. The offset was adjusted such that non-specific signals matched the secondary-only controls to ensure that only positive signals were displayed. Image files were saved as .CZI files given by the Zeiss Confocal Microscope software, ZEN. If fewer than five labels are used, the lambda scan and spectral unmixing are not required, and each channel can be optimized individually using standard confocal imaging protocols. MyoSight cannot analyze z-stacks.

### Manual analysis for comparison to MyoSight

For manual analysis of fiber-type and CSA, samples were prepared and stained as described above. Images were analyzed in FIJI using the free hand tool to encircle individual myofibers. Manual analyses of CSA, fiber-type, perinuclei, and central nuclei were performed for all images used in this study. The determination of accuracy of the image analysis programs examined in this study was based on comparisons of program-derived results with manually acquired results.

### Inter-user reliability

To test the reproducibility of MyoSight, four users with different levels of experience in these types of analyses were selected to analyze single images from WT and mdx soleus muscles. Users were asked to complete CSA, fiber type, and nuclei analysis using MyoSight with only the instruction manual as their guide (Supplemental Material).

### Statistical analyses

*T* tests were used to determine differences in fiber-type-specific CSA, perinuclei, and central nuclei between WT and mdx mice. To compare MyoSight to manual analysis, Pearson correlations were used on a subset of 20 fibers of each fiber-type as analyzed by either MyoSight or manual methods. To compare WT to mdx for binned CSA analysis, two-way ANOVAs were used with Sidak’s multiple *T* test comparisons between WT and mdx. Statistical significance was set at an alpha value of *p* < 0.05 for all methods.

## Results

### Overview and use of MyoSight

The MyoSight program functions as a plugin for FIJI, a freely available image analysis platform produced by the National Institutes of Health. The program, instruction manual, and test images are available on GitHub and are included in the Supplemental Material.

MyoSight uses a series of dialog boxes to guide users during analyses and provide control over the automated processes. Users are first prompted to choose an image type for analysis, either Bio-Format or “Other” if a TIFF or JPEG image is used. The descriptions in the remainder of this section are specific for Bio-Format files, but instructions for analysis of TIFF or JPEG files are provided in the Supplemental Material. Channel and fluorophore information are provided to allow each individual channel to be assigned to the correct fluorophore. Users are directed to select a folder in which to save the output data following image analysis. After selecting an image file for analysis, users are guided through a process to optimize detection of the laminin stain.

Adjustable parameters include “Prominence,” “Particle Size,” and “Threshold”. Prominence determines the degree of segmentation. Assignment of smaller values increases the sensitivity for the laminin stain but increases the risk of counting a single fiber as two fibers. Higher prominence values decrease sensitivity but increase the risk of inaccurate analysis of a weak laminin stain. “Particle Size” is the smallest CSA the program will recognize as a myofiber to exclude small intracellular spaces, blood vessels, and small tears from the cryo-sectioning process. Threshold determines the lower and upper limits of fluorescence signal. The default settings work well with optimal laminin staining and imaging and, in our analyses, gave the most accurate CSA. The user can modify these settings based on the quality of the laminin stain, but changes should be made with caution. For example, the “Huang” threshold type is more sensitive and can pick up weaker laminin stains but leads to smaller CSA measurements when the laminin stain is strong.

After the initial values are set, MyoSight defines fiber borders and creates a region of interest (ROI) corresponding to each individual fiber’s border. The accuracy of these ROIs can be checked, and users can delete and re-draw any incorrect ROIs using the freehand selection tool. Prominence and particle size values or threshold type can be adjusted, and the analysis repeated as needed. To designate muscle fiber-type, threshold values are set for each channel assigned to an MHC isoform, and the results are shown in a new window. Assigned fiber-types can be checked and corrected either by manually changing inaccurate fiber-type labels or by adjusting the threshold values and re-analyzing.

Once the fiber-type analyses are complete, MyoSight proceeds with central and perinuclei quantification. No user input is required for this step. When all analyses are complete, users can either select another image to analyze or end the program. At the completion of the analyses, all data, including myofiber CSA, Feret’s diameter, fiber type, number of central nuclei, and the number of perinuclei for each fiber are saved in a text file in the designated output folder. All annotated image files with designated ROIs and fiber types are also saved as TIFF files. If more images are analyzed, all channel assignments are applied to subsequent images. If the user selects the “Analysis Complete” option, MyoSight ends the program and closes all FIJI windows.

### Identification of fiber borders

**Fig. 1:**
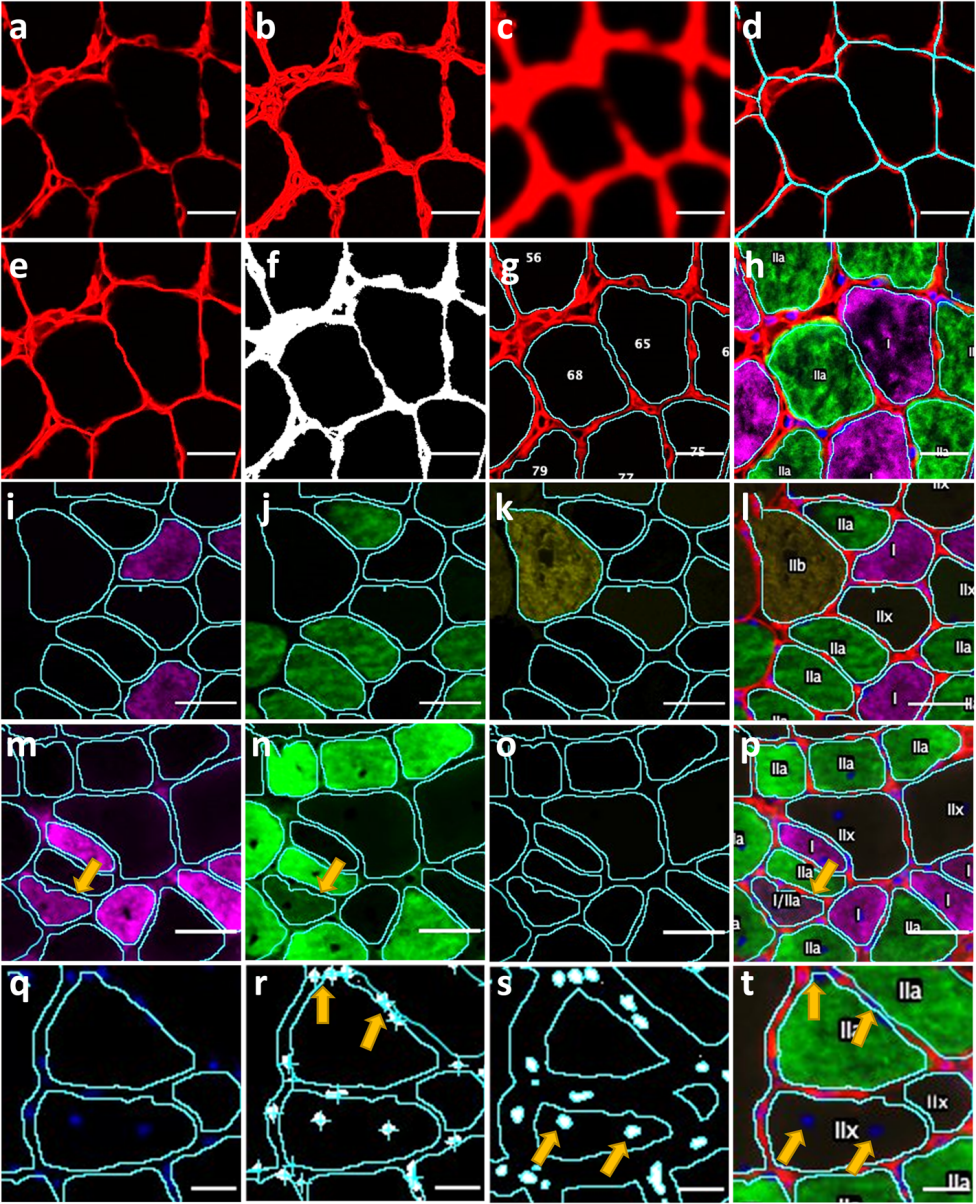
MyoSight identification of fiber borders, fiber-type, peri- and central nuclei. Representative images illustrating fiber border identification, fiber-type recognition, and peri- and central nuclei counting in the soleus from a WT mouse. **a** Representative image of original laminin stain in soleus of a WT mouse. **b** The FIJI “Find Edges” tool is used to enhance weak laminin staining. **c** Gaussian blurs are used to connect the breaks in the laminin staining separating adjacent fibers. **d** Fiber segmentation lines overlayed on original laminin stain. **e** Segmentation lines are colored to match laminin stain, and the image is flattened to enhance the laminin stain. **f** The flattened image is thresholded. **g** Individual regions of interest are created for each fiber. **h** All channels are combined for manual corrections of fiber borders and fiber-type. Representative images of fibers stained with antibodies to **i** MHC I, **j** MHC IIa, **k** MHC IIb, and **l** merged MHC immunofluorescent staining for all fiber-types in the soleus of a WT mouse with fiber borders overlaid. Representative MHC I/MHC IIa hybrid fiber defined by average pixel brightness for a fiber exceeds the threshold in two channels. **m** MHC I, **n** MHC IIa, **o** MHC IIb, and **p** merged. MHC immunofluorescent staining for all fiber types in the soleus of a WT mouse with fiber borders overlaid. Arrows indicate a hybrid fiber. **q** Representative images of nuclei staining in the soleus of an mdx mouse with fiber borders overlaid. **r** Perinuclei counting. The nuclei stain is subjected to watershed segmentation with a cross placed over the centroid of each nuclear region. Arrows indicate nuclei whose centroid is inside the fiber border and counted as fiber specific perinuclei. **s** Central nuclei counting. The fiber regions are reduced in size to include only the central region of each fiber. Arrows indicate nuclei whose regions overlap with the central region of a fiber and counted as a centralized nuclei. **t** Representative images of all MHC immunofluorescent staining and nuclei staining with fiber borders overlaid. Arrows indicate peri- and central nuclei from panel **r** and panel **s**, respectively. Scale bars are 20 microns (**a**–**h**), 40 microns (**i**–**p**), and 20 microns (**q**–**t**)

The algorithm used by MyoSight to identify fiber borders uses a combination of segmentation and thresholding, as illustrated in Fig. 1. The “find edges” command, Gaussian blurs, and contrast enhancements are used to enhance weak membrane staining between adjacent myofibers to allow for accurate segmentation (Fig. 1a–d). The segmentation lines are re-colored and overlaid on the original laminin stain to identify borders between adjacent fibers that are weakly stained. The segmentation lines are then thresholded along with the original membrane stain for a defined ROI corresponding to each fiber (Fig. 1e–g). Even without manual corrections, MyoSight accurately identifies membrane borders of fibers with poor immunofluorescent laminin staining (Fig. 1h).

### Identification of fiber-type

Identification of fiber-type is performed by splitting the merged image into individual channels and overlaying the ROIs generated from the laminin stain with the fiber-type stains in their respective channels. MyoSight then determines whether the average pixel brightness within that region exceeds a threshold given by the user for each fiber-type (Fig. 1i–k). The images for laminin and each fiber-type are then merged with an overlay of all defined fiber-types (Fig. 1l). Using the staining protocol detailed here, myofibers that did not meet the threshold criteria are categorized as MHC IIx fibers since no primary antibody was used to stain for IIx fibers. If the average pixel brightness for a fiber exceeds the threshold in two channels, it is designated as a hybrid fiber (Fig. 1m–p).

### Identification of peri- and central nuclei

Fiber perinuclei are identified by whether the centroid of each nucleus is within a fiber’s border. First, the DAPI stain is overlaid with the defined fiber ROIs (Fig. 1q). The DAPI stain is then thresholded and subjected to a watershed segmentation to ensure two adjacent nuclei are not counted as one. Next, the centroid of each nucleus is determined and assessed for whether it exists within the borders of a myofiber (Fig. 1r). Central nuclei are identified by reducing the size of the ROIs for each myofiber to exclude the fiber border (Fig. 1t). The reduction factor is based on the average size of myonuclei and ensures that larger perinuclei are not counted as central nuclei. The ROIs generated from the initial watershed segmentation of the DAPI stain are each assessed to determine if it is located in the central region of each myofiber (Fig. 1t).

### Manual correction of common errors

**Fig. 2:**
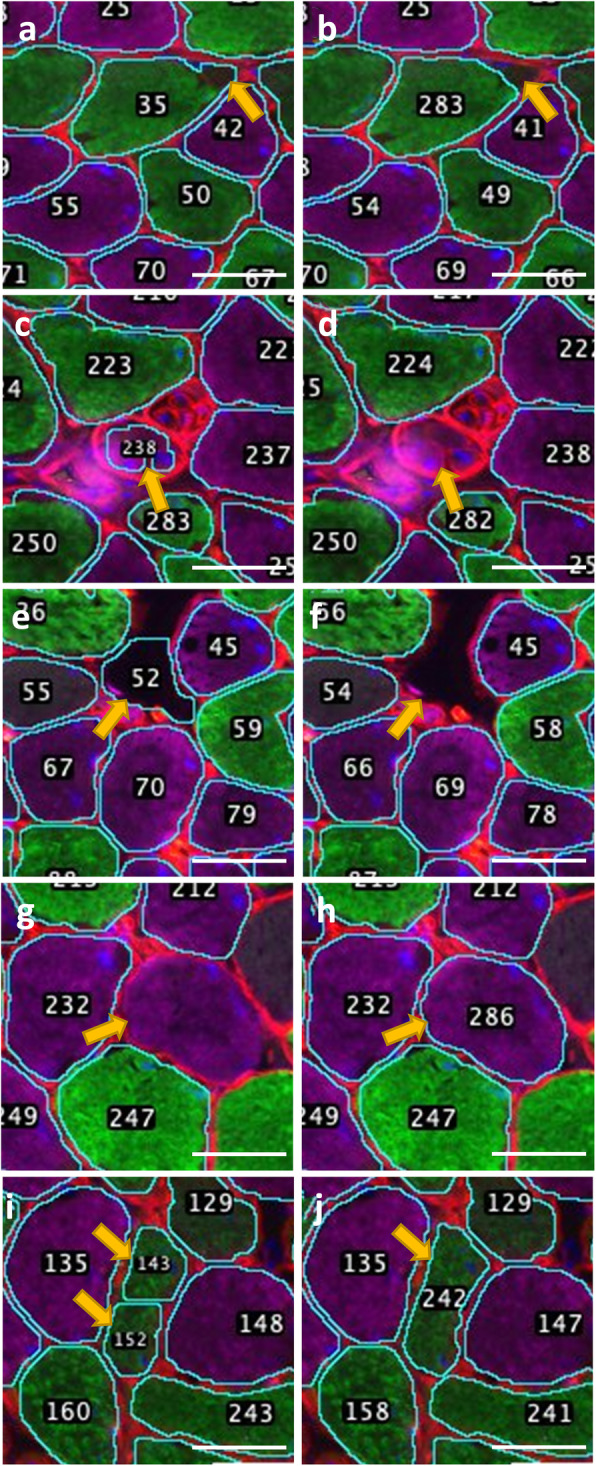
Manual corrections. **a** Representative image of incorrect analysis (Fiber 35) due to a discontinuation of laminin staining along the fiber border (arrow). **b** Manual correction of incorrectly defined fiber border in panel **a** (fiber 283). **c** Representative image of a muscle spindle (designated in this analysis as fiber 238) incorrectly identified as a myofiber. **d** Manual correction of incorrectly identified muscle spindle in panel **c**. **e** Representative image of interstitial region incorrectly identified as a myofiber (designated in this image as fiber 52). **f** Manual correction of incorrectly identified region in panel **e**. **g** Representative image of myofiber that was not identified. **h** Manual correction of unidentified myofiber in panel **g** (fiber 286). **h** Representative image of a single myofiber incorrectly identified as two fibers (designated 143 and 152 in this image). **j** Manual correction of incorrectly identified myofiber from panel (now fiber 242). Scale bars are 40 micron

No image analysis program provides perfect analyses every time. The goal of an image analysis program should be to minimize, but not eliminate, user input. Manual corrections are frequently needed to redraw incorrect ROIs and optimize the parameters that control ROI assignment. The most common cause for incorrect ROIs is a break in laminin staining along the fiber border or a separation between the myofiber border and its membrane due to freeze artifact (Fig. 2a). The fiber border in this example needs to be deleted and re-drawn (Fig. 2b). Another type of incorrect ROI occurs when a blood vessel, muscle spindle, or Golgi tendon organ with surrounding laminin stain is segmented and misidentified as a muscle fiber (Fig. 2c). These ROIs should be deleted (Fig. 2d). Another common incorrect ROIs occurs when the interstitial space between fibers is misidentified as a fiber (Fig. 2e) This type of misassignment frequently occurs when there is a tear or hole in the cross section leading to a large empty space or when the value for particle size is set too low and smaller regions between fibers are segmented. These incorrect ROIs should also be deleted (Fig. 2f). Another correction that is sometimes needed is for missing ROIs (Fig. 2g). This requires that the ROI be redrawn (Fig. 2h). The last type of incorrect ROI occurs when a single fiber is incorrectly segmented into two (Fig. 2i). This can occur if the prominence value is set too low and is corrected by adjusting the prominence value and re-analyzing, or by deleting and re-drawing the ROI (Fig. 2j)**.**

### Comparisons to other freely available image analysis programs

Several programs have been developed to facilitate fast, automated CSA and fiber-type analysis. Some of the more commonly used are Muscle J [6], Open-CSAM [10], MyoVision [8], and SMASH [11]. We developed MyoSight to combine useful features available separately in other programs. MyoSight is semi-automated, allowing for manual corrections as well as reanalysis with new analysis parameters chosen by the user to optimize the analysis algorithm. MyoSight operates in conjunction with FIJI and is available on both Mac and PC operating systems to maximize accessibility. The use of multi-channel Bio-Format images streamlines the analysis process, requiring import of only a single file which has embedded scaling information. Using the staining and imaging protocols detailed here (Supplemental Material), MyoSight can acquire CSA and fiber-type information for four fiber-types, central nuclei, and perinuclei. These results are automatically saved in a text file that can be imported to the relevant graphing and statistical software. Additionally, MyoSight provides images of all analyses including CSA, fiber-type, perinuclei, and central nuclei, allowing the user to demonstrate the accuracy of their measurements. A comparison of the features of MyoSight and other currently available programs is presented in Table 2.

**Table 2:** **Characteristics of Freely Available Image Analysis Programs**

	MyoSight	MuscleJ	Open-CSAM	MyoVision	SMASH
**Compatibility**	PC/Mac	PC/Mac	PC/Mac	PC Only	PC Only
**Platform**	FIJI	FIJI	FIJI	Open Source	MATLAB
**Image type**	Original format/TIFF	Original format/TIFF	TIFF	TIFF	TIFF
**Auto/semi-automated**	Semi-automated	Automated	Semi-automated	Automated	Semi-automated
**Manual corrections**	+	−	+	−	+
**Re-analysis with new analysis parameters**	+	−	−	−	+
**CSA measurements**	+	+	+	+	+
**Identifies fiber-types**	+	+	−	+	+
**Identifies perinuclei**	+	+	−	+	−
**Identifies central nuclei**	+	+	−	−	+
**Identifies satellite cells**	−	+	−	−	−
**Identifies vessels**	−	+	−	−	−
**Accepts Z-stacks**	−	+	−	−	−
**Number of channels**	5	4	1	5	3
**Result files automatically generated**	+	+	−	+	+
**Output of analyses performed**	+	+	−	−	+

Side-by-side comparisons of current freely available image analysis programs. Comparisons made include the computer operating software compatibility requirements, platform used to carry out the functions of the program, image type that can be used, analysis capabilities, and the type of output of results.

### Inter-user reliability

**Fig. 3:**
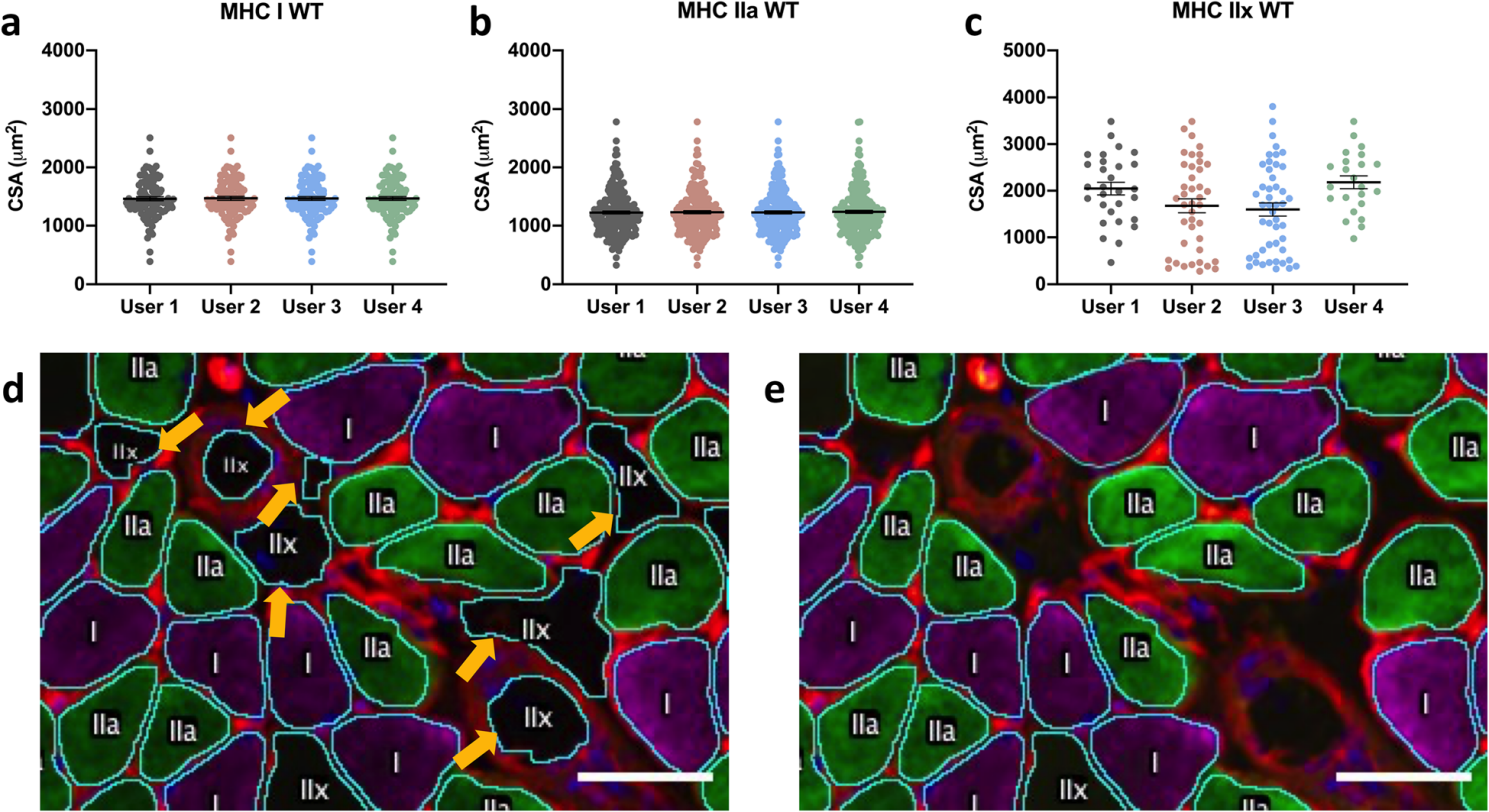
Inter-user reliability. Independent analyses of CSA as a function of fiber type by 4 users. Data from soleus of a WT mouse. **a** CSA of MHC I fibers in WT soleus, *p* > 0.05. **b** CSA of MHC IIa fibers from WT *p* > 0.05. **c** CSA of MHC IIx fibers from WT, *p* = 0.047. **d** Analyzed merged WT image from user 3. (Arrows indicate structures misidentified as fibers.) **e** Analyzed merged WT image from user 4. Scale bars are 50 microns

To assess the ease and accuracy of analyses with MyoSight, we asked four individuals in the laboratory to analyze the CSAs of different fiber-types from a single image from WT soleus and a single image from the mdx soleus. While the analyses of the CSA of MHC I and IIa fibers were similar among the four users (Fig. 3a, b), the analyses of MHC IIx fibers showed significant variability (Fig. 3c). The output of the analyses from users 3 and 4 (Fig. 3d and e, respectively) shows the reason for the variability. User 3 incorrectly identified several spaces between fibers as fibers (arrows in Fig. 3d). This emphasizes the need for the post-automated analyses manual corrections and indicates that some instruction and practice are required for new users to correctly identify muscle fibers and use the program. This further demonstrates the importance of the image outputs of the analyses taken by MyoSight to check the accuracy of the analysis, and can also be used as an important instructional tool.

### Comparison between WT and mdx solei analyzed by MyoSight and manual methods

**Fig. 4:**
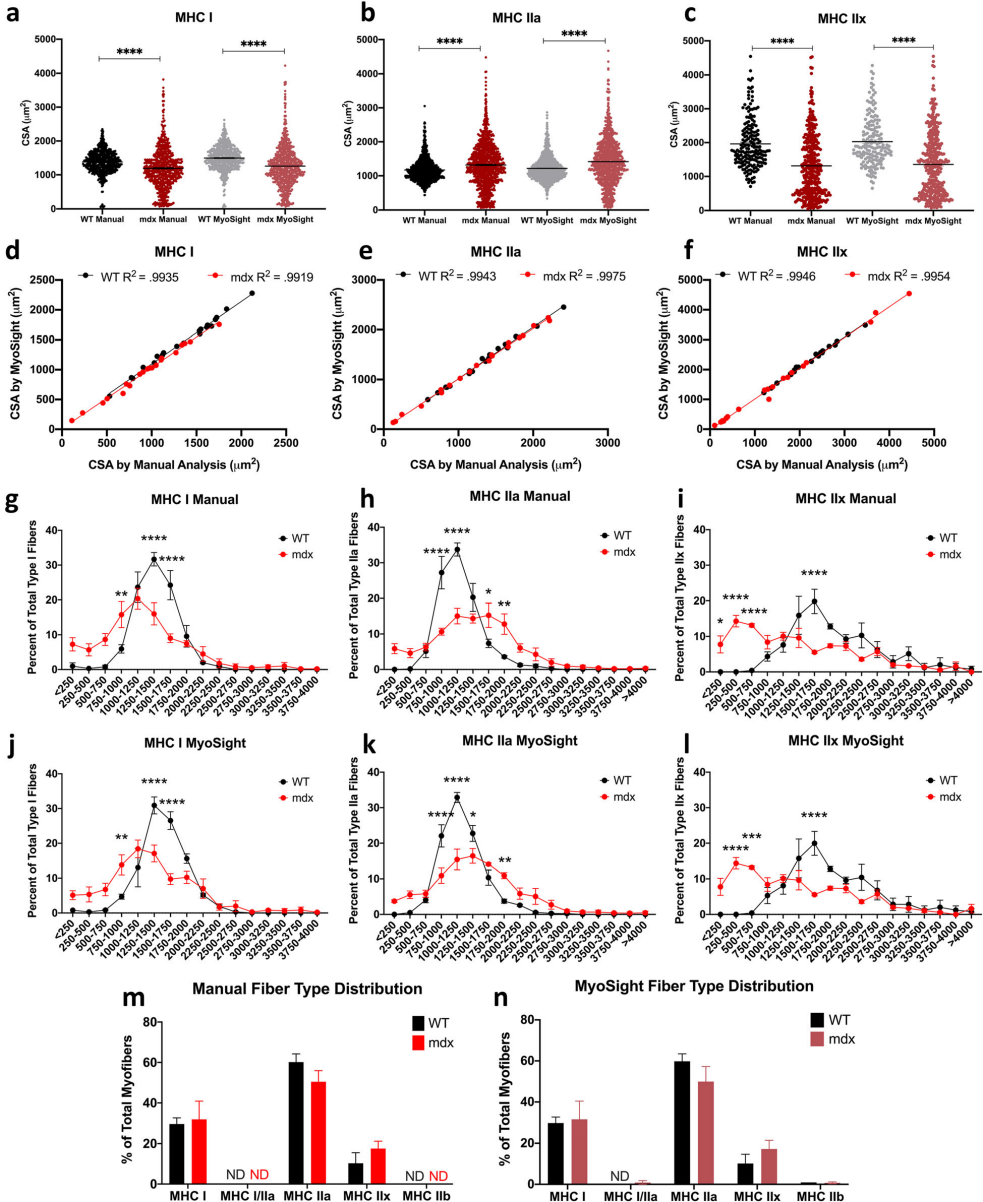
CSA, fiber-type specific CSA, and fiber-type distribution. Comparisons of manual analysis to the analyses from MyoSight. **a** MHC I fibers from 3 WT and 3 mdx mice analyzed either manually (WT: *n* = 574 fibers, mdx: *n* = 572 fibers) or with MyoSight (WT: *n* = 664 fibers, mdx: *n* = 657 fibers). **b** MHC IIa fibers from 3 WT and 3 mdx mice analyzed either manually (WT: *n* = 1155 fibers, mdx: *n* = 1165 fibers) or with MyoSight (WT: *n* = 1039 fibers, mdx: *n* = 968 fibers). **c** MHC IIx fibers from 3 WT and 3 mdx mice analyzed either manually (WT: *n* = 193 fibers, mdx: *n* = 178 fibers) or with MyoSight (WT: *n* = 358 fibers, mdx: *n* = 358 fibers). **d** Correlation of CSA of MHC I fibers from WT and mdx mice from MyoSight with the CSAs of these fibers assessed manually. **e** Correlation of CSA of MHC IIa fibers from WT and mdx mice from MyoSight with the CSAs of these fibers assessed manually. **f** Correlation of CSA of MHC IIx fibers from WT and mdx mice from MyoSight with the CSAs of these fibers assessed manually. **g** Distribution of type I fibers analyzed manually as a function of binned CSA for WT and mdx fibers (*n* = 3, mean ± SEM). **h** Distribution of MHC IIa fibers analyzed manually as a function of binned CSA for WT and mdx fibers (*n* = 3, mean ± SEM). **i** Distribution of MHC IIx fibers analyzed manually as a function of binned CSA for WT and mdx fibers (*n* = 3, mean ± SEM). **j** Distribution of MHC I fibers analyzed with MyoSight as a function of binned CSA for WT and mdx fibers (*n* = 3, mean ± SEM). **k** Distribution of MHC IIa fibers analyzed with MyoSight as a function of binned CSA for WT and mdx fibers (*n* = 3, mean ± SEM). **l** Distribution of type IIx fibers analyzed with MyoSight as a function of binned CSA for WT and mdx fibers (*n* = 3, mean ± SEM). **m** Fiber-type distribution determined from manual analysis (*n* = 3). **n** Fiber-type distribution determined by MyoSight analysis (*n* = 3). ND, not detected. Differences between WT and mdx represented by * *p* ≤ 0.05,** *p* ≤ 0.01,*** *p* ≤ 0.001, and **** *p* ≤ 0.0001

As mentioned previously, a major goal for creating a new program was to accurately identify differences in fiber size, fiber-type distribution, perinuclei, and central nuclei that arise from pathogenic processes associated with muscle disease. To illustrate the ability of MyoSight to detect disease-related differences, we analyzed WT and mdx solei using MyoSight and manual analyses. Since pathological changes in skeletal muscle often occur in a fiber-type-dependent manner, we divided all data by fiber type. While MyoSight is able to detect MHC IIB, the extremely low number of these fibers in the soleus precluded statistical analyses. In Fig. 4a–c, we illustrate the variations in CSAs for MHC I, IIa, and IIx fibers, respectively, in solei from 3 WT and 3 mdx mice as detected by manual and MyoSight analyses. From these plots, it can be seen that the mdx mice display an increase in both very small and very large fibers of all 3 fiber types compared to the fibers from the solei of WT mice. CSA measurements from MyoSight display highly significant correlations with the data obtained from manual analyses for all fiber types (Fig. 4d–f). While Fig. 4a–c combine CSA data from all fibers analyzed from the solei of 3 mice of each genotype, we have also compared the percent of the total fibers of each type per mouse in soleus sections in binned fiber size groups (Fig. 4g–l). Again, the dramatic and highly significant changes in the distributions of CSAs of MHC I, IIa, and IIx fibers in the solei of the mdx mice compared to WT fibers are clearly detected both with manual methods (Fig. 4g–i) and MyoSight (Fig. 4j–l). MyoSight also performs comparably to the manual analyses for the identification of changes in fiber-type distribution (Fig. 4m and n). However, higher *n* numbers are required to reach significance for this type of analysis.

**Fig. 5:**
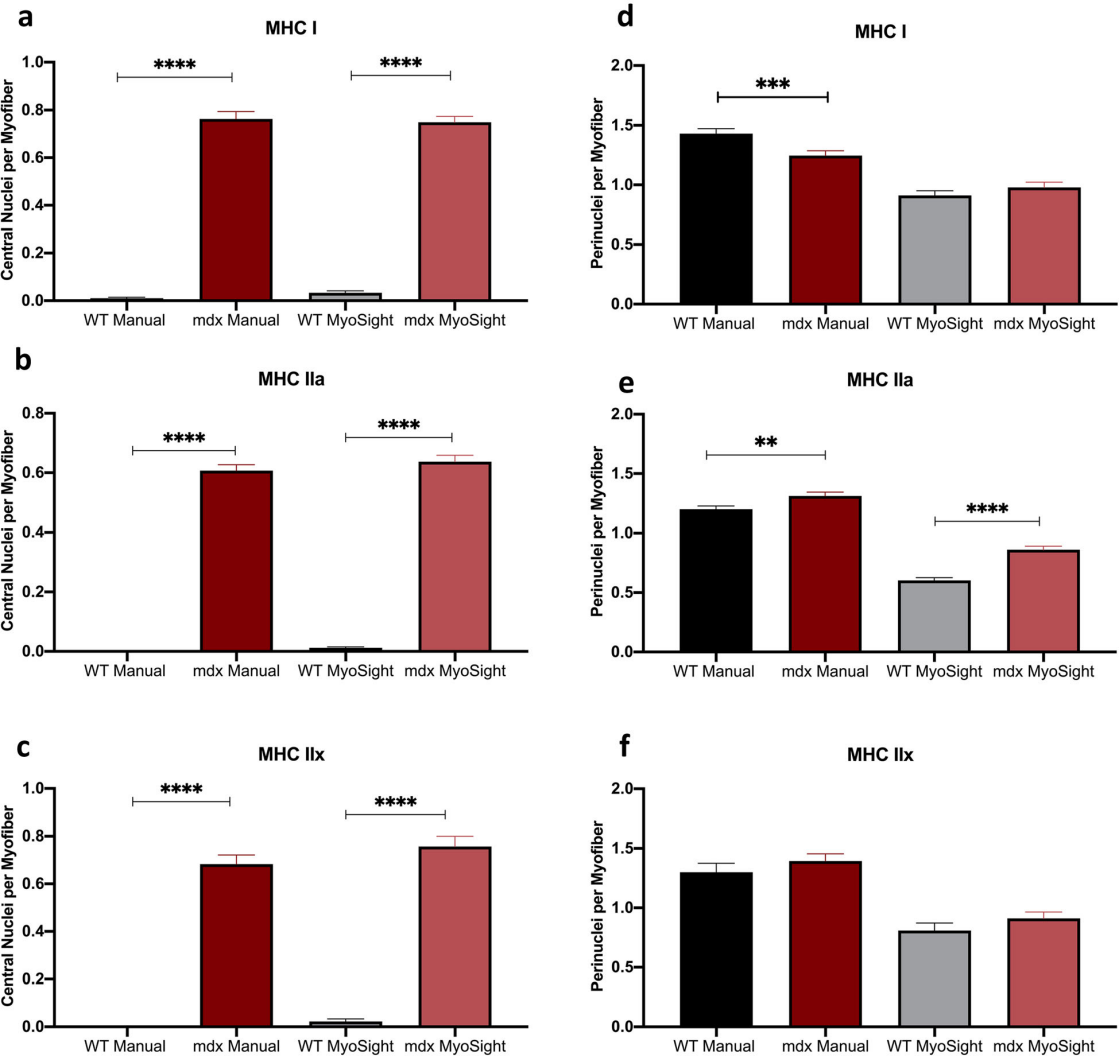
Analyses of central and perinuclei in I, IIa, and IIx for WT and mdx mice. **a** The number of central nuclei in type I fibers from WT and mdx mice determined manually and with MyoSight. **b** The number of central nuclei in type IIa fibers from WT and mdx mice determined manually and with MyoSight. **c** The number of central nuclei in type IIx fibers from WT and mdx mice determined manually and with MyoSight. **d** The number of perinuclei in type I fibers from WT and mdx mice determined manually and with MyoSight. **e** The number of perinuclei in type IIa fibers from WT and mdx mice determined manually and with MyoSight. **f** The number of perinuclei in type IIx fibers from WT and mdx mice determined manually and with MyoSight. Differences between WT and mdx represented by * *p* ≤ 0.05,** *p* ≤ 0.01,*** *p* ≤ 0.001, and **** *p* ≤ 0.0001

Two other important parameters needed to assess the consequence and treatments of injury and disease of skeletal muscle are the numbers of centralized and perinuclei in different fiber-types in muscle damage/repair [12, 13], and muscle diseases such as centronuclear myopathy (CNM) [14–16] which lead to centralized nuclei. The muscle of mdx mice also displays an increase in centralized nuclei [17], and this is clearly detected in MHC I, IIa, and IIx fibers from the solei mdx compared to WT mice using both the manual and MyoSight programs (Fig. 5a–c). One issue with determining the number of perinuclei per fiber is distinguishing between nuclei associated with cells outside the muscle membrane (for example, closely associated myoblasts) and the nuclei that are located within the muscle, just under the sarcolemmal membrane. This is visually very difficult with the manual approach. Manual analyses likely detect more “perinuclei” than MyoSight (Fig. 5d–f). When assessing perinuclei, MyoSight removes user bias by applying defined criteria for perinuclear identification and employs these criteria consistently for all analyses.

## Discussion

To fully delineate the consequences of a muscle disease, aging, or injury to muscle and assess efficacy of interventions, it is critical to analyze muscle cross-sectional area in a fiber-type-specific manner, determine if fiber-type distributions have changed, and assess the number of central and peripheral nuclei. Our goal in the development of MyoSight was to create an accurate algorithm that is user friendly and requires minimal post-analysis corrections. MyoSight’s initial segmentation and thresholding processes to identify membrane borders compares favorably to other programs. The accuracy in the quantification of CSA and fiber-type distributions is enhanced by MyoSight’s inclusion of user input in the algorithm and the ability to reanalyze with new input to further optimize the algorithm. The visual outputs from MyoSight also offer opportunities to educate inexperienced users to improve their accuracy in analyses of CSA and fiber-type distributions.

A number of outstanding programs to analyze muscle CSA and fiber type are available to muscle researchers. All of these programs offer users advantages when compared to manual analysis. SMASH was one of the first freely available image analysis programs designed for skeletal muscle cross sections, offering CSA measurements, central nuclei, and up to two fiber types and a mask or all the ROIs generated [11]. MyoVision added the ability to analyze all fiber-types and perinuclei [8]. Open-CSAM added the ability to manually correct inaccuracies before saving data [10]. SMASH and MyoVision use TIFF images which can be opened by most image processing applications and may be the preferred programs if acquisition software that produces BioFormat images is not available. MuscleJ, which provides rapid analyses, is capable of analyzing satellite cells and blood vessels in addition to fiber-type and nuclei, and offers a visual output of the analysis [6]. MuscleJ is likely to be the preferred program when very large numbers of images need to be analyzed. We sought to enhance the user experience and accuracy by combining many of these features to allow the user to optimize the algorithm for each image and correct any inaccuracies for analysis of skeletal muscle fiber CSA, all fiber types, perinuclei, and central nuclei.

## Conclusions

We present a new semi-automated program, MyoSight, for analyses of muscle cross-sectional area, fiber-type distribution, number of perinuclei, and number of centralized nuclei. This program combines multiple features previously seen in other programs in a way to maximize accuracy and transparency in the data while drastically reducing analysis time compared to manual analysis. MyoSight is designed to improve the analyses of skeletal muscle cross sections needed to assess the consequences of disease and therapeutic interventions.

## Supplementary information

**Additional file 1.** Supplementary materials– 1. MyoSight Program. 2. Instruction Manual, Supplementary materials 3. Test image 1. Supplementary materials 4. Test image 2

## Data Availability

The program, instruction manual, test images, and all data generated are readily available and/or included in this article and/or supplemental information and readily accessible online sites.

## Abbreviations

MHC: Myosin heavy chain
CSA: Cross-sectional area
PBS: Phosphate-buffered saline
DAPI: 4′,6-diamidino-2-phenylindole
IF: Immunofluorescent
OCC: Optimal cutting compound
SMASH: Skeletal muscle analysis by segmented histology
ROI: Region of interest
mdx: Mouse model of Duchenne muscular dystrophy
WT: Wild type
